# The Cybersecurity and the Care Robots: A Viewpoint on the Open Problems and the Perspectives

**DOI:** 10.3390/healthcare9121653

**Published:** 2021-11-29

**Authors:** Daniele Giansanti, Rosario Alfio Gulino

**Affiliations:** 1 Centre Tisp, Istituto Superiore di Sanità, 00161 Rome, Italy; 2 Faculty of Engineering, Tor Vergata University, Via Cracovia, 00133 Roma, Italy; rosario.gulino.uni.tv@hotmail.com

**Keywords:** e-health, medical devices, m-health, rehabilitation, robotics, organization models, artificial intelligence, electronic surveys, social robots, collaborative robots, cyber security, cyber risk, informatics

## Abstract

Care robots represent an opportunity for the health domain. The use of these robots has important implications. They can be used in surgery, rehabilitation, assistance, therapy, and other medical fields. Therefore, care robots (CR)s, have both important physical and psychological implications during their use. Furthermore, these devices, meet important data in clinical applications. These data must be protected. Therefore, cybersecurity (CS) has become a crucial characteristic that concerns all the involved actors. The study investigated the collocation of CRs in the context of CS studies in the health domain. Problems and peculiarities of these devices, with reference to the CS, were faced, investigating in different scientific databases. Highlights, ranging also from ethics implications up to the regulatory legal framework (ensuring safety and cybersecurity) have been reported. Models and cyber-attacks applicable on the CRs have been identified.

## 1. Introduction

The cybersecurity (CS) in healthcare deals with the cyber risks in the cyber-systems used in the *health domain*. These systems can be medical devices and/or a complex interoperable and heterogeneous systems (e.g., Radiology Information System) [1,2]. A frightening growth is expected in the sector of the care robots (CR)s. The applications of social robots [3,4], for example, are continuously increasing [5,6].

Hence, it is now very important to address CS in CRs.

The Policy Department for Economic, Scientific and Quality of Life Policies, of the European Parliament, identified the most interesting applications for the CRs [7]: *Robotic surgery, Care and Socially assistive Robots, Rehabilitation systems, Training for health and care workers*. The sector is wide, complex and with numerous implications for the CS. For example, the rehabilitation robotics [8] has three motion applications (Table 1):

1. The stability.
2. The lower limbs.
3. The upper limbs.

Furthermore, rehabilitation robots use two different technological solutions (exoskeleton technology and end-effector technology), with different implications for the CS.

Social robots (SR)s are used in several diversified fields of assistance and rehabilitation [3,4]. Similar considerations can be carried out for the other applications. The implications between technologies, applications and CS immediately emerge from the definition of CR. CRs are complex and interoperable systems [9]. The European Foresight Monitoring Network [10] defines the CR as a system “able to perform coordinated mechatronic actions (force or movement exertions) based on processing information acquired through sensor technology, to support the functioning of impaired individuals, medical interventions, care and rehabilitation of patients and also individuals in prevention programs”.

The European Parliament traced for the CR the direction of the CS, highlighting that (literally cited) “possible applications of AI and robotics in medical care (are) managing medical records and data, performing repetitive jobs (analysing tests, X-rays, CT scans, data entry), treatment design, digital consultation (such as medical consultation based on personal medical history and common medical knowledge), virtual nurses, medication management, drug creation, precision medicine (as genetics and genomics look for mutations and links to disease from the information in DNA), health monitoring and healthcare system analysis, among other.” [11].

It is important to investigate the progress of CS studies on the CRs. It is also important to investigate the problems and peculiarities. Correlations with other disciplines are important, such as, for example, ethics and regulation.

CRs, in fact, have characteristics, that are not found on other devices. They can replace caregivers or provide psychological or motor rehabilitation. The implications of CS in a programming error or a sabotage are high. Traditional problems can be found. However, many others are added. Motor damage can occur. Psychological damage can occur. Think about the false relationship that can be created with a pet SR. Think about the problems that an incorrect programming of the ethics concepts of an SR can bring.

The objective of the study is:

(a) To investigate the positioning of CRs in CS studies.
(b) Analyse the problems and peculiarities of the devices that have an impact in this area.
(c) Take stock of the related issues of ethics and regulation.

In this paper the authors discuss the conception of a viewpoint, presented and explained in four sections (plus the introduction and conclusions).

The first section (paragraph 2: The position of the care robots in the studies) deals with the state of production of studies in this area. This is carried out through an analysis of the production of scientific literature. The second section (paragraph. 3: Ethics, care robots and cybersecurity) deals with the impact of the ethical issues. In particular, the correlation of the CS both with the ethics of research and with the programming of ethics on CRs is highlighted. The third section (paragraph 4: Regulatory framework, care robots and cybersecurity) deals with the situation of the regulatory framework. The fourth section (paragraph. 5: Cyber-attacks applicable to care robots) reports models and cyber-attacks.

## 2. The Position of the Care Robots in the Studies

We are certainly witnessing a growing interest in the CS.

A simple search on the Pubmed database, the most important database of the health domain, shows 12.785 results on the cyber security [12]. Among them, a group identified in [13] deals with robots. By expanding the search with the keys safety and risk we find:

4882 articles with the key (safety [Title/Abstract]) AND (robot) [14].

5005 articles with the key (risk [Title/Abstract]) AND (robot) [15].

Scientists refer to safety or risk also to address issues related to informatic faults/problems. These informatic problems/faults can affect the mechatronics, and therefore, the human interface. This is a CS issue. Certainly, this is a first important indication for scholars. The experience gained in the sector in the industry, production, and consuming sector (IPCS) is another important issue to consider. Here, the theme of the safety of robot-human interaction in the workplace is highly developed. Here, the topic has been dealt with for much longer. Safety in robots is addressed. However, the use of robots for security is also addressed. Both are CS related issues. Part of the experience gained here, can be exported and readapted in the health domain, a particular workplace. Presently [16], there are three categories of robots in the IPCS: (1) industrial robots; (2) professional and personal service robots, and (3) collaborative robots. Studies reporting recommendations are spreading for these types of robots [16,17]. Some studies are specifically dealing with physical security [18] also in relation to CS. Other studies are dealing with traditional issues, such as security and privacy issues [19].

Very interesting models dealt with the security in the workplace. The Advanced Human-Robot Collaboration Model (AHRCM) approach was proposed in [20]. The idea was to enhance the risk assessment and to improve the safety in the workplace. The experimental results showed that the proposed AHRCM model achieved high performance in human-robot collaboration to reduce the risk.

The recent review in [21] highlighted how CS experience in IPCS robotics is exportable to the world of CRs. The same authors highlighted models and types of cyber-attacks on the CRs. Recent studies dealt with the security with SRs [22]. This included: risk assessment of communications security, predictive analysis of security risks, implementing access control policies to enhance the security of solution, and auditing of the solution against security, safety and privacy guidelines and regulations. A limited approach to some issues of CS was addressed in a few studies, such as in surgical applications [23] or in the rehabilitation of the lower limbs [24].

Other studies showed a backwardness in importing into the health domain the experience made elsewhere [25]. Probably, this is due to the limits and inadequacy of legislation concerning the CS [9,26]. It is also very important to observe how scientific societies move around the CS theme.

For example, CS has now become an indispensable issue in the topic Human Computer Interaction (HCI), in international scientific meetings [27]. In fact, one of the most important international conferences on HCI, hosts a section (HCI-CPT: International Conference on HCI for Cybersecurity, Privacy and Trust) dedicated to the CS applied to HCI. This highlights the importance of the theme for machines that interface/integrate with the human. In [28], a work presented at the HCI-CPT, it is also highlighted how the analysis must be extended directly in the field (for example in the workplace), involving the insiders in targeted investigations, with dedicated surveys, to understand behaviours at risk, as regards CS.

It is also necessary to consider the peculiarities of the CRs.

The ethical implications for the CRs are much more relevant than for other categories of robots. It is also necessary to consider more risks and criticalities. These risks and criticalities affect not only the physical issues, but also the psychological issues [9].

It was proposed in [9] a model describing the relationships between cyber-attacks/software fault/AI deficit and the impact on human safety.

**Figure 1:**
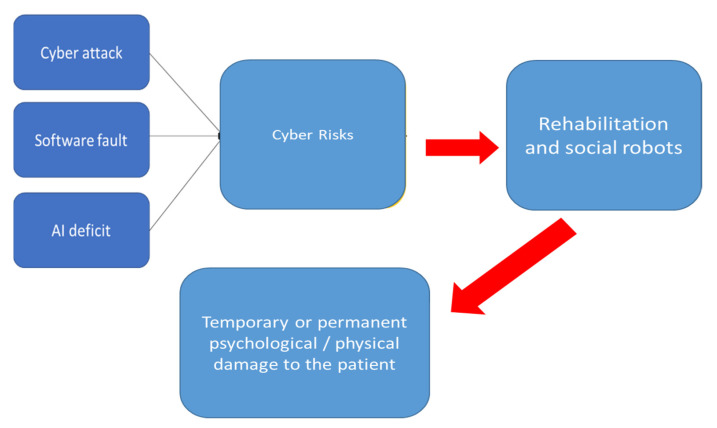
Model of health risks for the CRs.

We specialize in Figure 1, the model in the case of rehabilitation and assistance robotics. This model highlights the health risks for the user.

## 3. Ethics, Care Robots and Cybersecurity

Very important ethical discussions are open. A search on Pubmed with the key (social robot) AND (ethics) shows some interesting scientific contributes [29], confirming the wide discussion around the ethics. Ethics has a strong impact on the world of the CRs. This is reflected in the CS. We extended here the search also to other databases.

We can undoubtedly distinguish two important macro-sectors with an impact on CS. The first macro-sector is the ethics in a responsible research and innovation [30]. The second macro-sector is the ethics problem encountered while building moral CRs [31].

Stahl and Coeckelbergh highlighted, for the first macro-sector [30], that traditional approaches to the ethics of robotics are often distant from innovation practices and contexts of use. They listed in their review key concerns of ethics. As it has been highlighted in [30] there is a strong scientific production of ethics of CRs [32,33,34,35,36,37,38,39], or machine (medical) ethics [40,41,42,43,44] connected to the CRs. Three aspects were identified in [30].

First, there are important impacts both in the society and in the health domain:

Replacement and its implications for labour.

Replacement and its implications for the quality of care; they are the so-called de-humanisation and ‘‘cold’’ care.

Second, there are issues raised by human–robot interaction in the health domain and especially by the robot taking over tasks from humans, for instance: autonomy (connected to the implication of the robots take decision with autonomy) Role and tasks (connected to the changes in the workflow), Responsibility (connected to the responsibility chain in case of problems), The Deception (connected, for example, to the use of SRs as ‘social companions, related to questions of opportunities and justification). Trust (connected, for example, to the reliability of giving subjects (also frail) in the hands of a CR.

Third, there are issues traditionally connected to the CS as for example:

Privacy and data protection.

Safety and avoidance of harm.

The second macro-sector [31] on the ethics problems is encountered while building moral CRs. It focuses on the interdisciplinary field of machine ethics—that is, how to program ethical rules and concepts inside on a robot [45]. This sector has become of utmost importance because the recent technological developments in the field of the CRs and artificial intelligence in general [46,47,48,49,50]. Gordon highlighted that to make ethics [31] “computable” (literally cited “depends in part, on how the designers understand ethics and attempt to implement that understanding in programs, but also more generally on their expertise in the field”.

Based on the review [31] it was found that, scholars in the field in informatics applied to machine ethics have gaps in training and practical knowledge of ethics. There is therefore an important CS due to this.

From the previous analysis, a strong connection emerges between ethical issues and CS in the CRs. There is a strong need to rethink a more expanded CS also connected to the ethics in robotics.

## 4. Regulatory Framework, Care Robots and Cybersecurity

Surely when we consider the regulatory issues, we must ponder that CRs also use eHealth [51]. However, many other issues must be considered [9,26]. These issues range from the impact of mechatronics up to the use as a networked medical device. Some studies have highlighted lights and shadows of the regulatory framework [9], arranged in Europe into:

- Safety regulations [52].
- Legislation on medical devices (MD)s classification [53].
- Legal frameworks on the cybersecurity [54,55].

### 4.1. Care Robots and Safety Regulations

Robots, in general, and CRs, follow [52] the General Product Safety Directive (Directive 2001/95/EC of the European Parliament and of the Council of 3 December 2001 on general product safety 2001) and the Directive 85/374/EEC on liability for defective products. The applicability of product liability regulations is not easily and directly applicable in the context of robotics applications.

### 4.2. Care Robots and Medical Device Regulation

CRs, based on their destination of use, can be classified as a medical device (MD). The European Medical Device Regulation (Regulation (EU) 2017/745) [53] contains a detailed definition of MDs.

The Regulation contains three important actions (lights) in the direction of the CS concerning the minimization of the risks, the design of the software (including CS), the inclusion of the respect of a set of IT requirements also related to the CS.

The regulation [53] certainly presents great innovations for the CS. However, there are some shadows. The first shadow is that this regulation focuses a lot on manufacturers and little on recipients/users [9,26], who have a leading role. Perhaps, instruction sheets and manuals are not always enough. The second shadow [9,26] is that compliance with CS requirements is challenging, in part due to the potential overlap of different certification schemes with varying geographical or product scope and evolution of external regulations (see for example the Cybersecurity Act). The third shadow, we personally think applicable is that the intended use and certification must be aligned [8] and this it is not always easy to detect.

### 4.3. Care Robots and Regulations on the Cybersecurity

Three are the documents regarding the legal frameworks regulating CR CS [54,55,56]:

1. The directive on security of network and information systems (also called NIS Directive) that provides measures for boosting the overall CS in the EU [54].
2. The General Data Protection Regulation (GDPR) obligating to implement appropriate measures to ensure a level of security appropriate to relevant risks [55].
3. The EU Cyber-security Act (Regulation (EU) 2019/881) which establishes an EU-wide cybersecurity certification framework [57].

None of the documents has been specifically designed for CRs.

The first two documents [53,54] work in synergy.

According to the NIS Directive, operators need to respond appropriately to manage the CS in a network [9]. A Network can, (according to the NIS Directive [54]), include MDs, such as robots. As the healthcare providers also process personal data, they are, therefore, subject to the provisions of the GDPR [55].

The third document, the EU cybersecurity Act establishes a road map for voluntary CS certifications valid in the EU [56].

Among the evident limitations of the three documents [54,55,56] (in addition to the fact that they are not specifically designed for CRs) we find that: the first two delegate CS to healthcare providers, although they can be found on the market CRs with very different levels of CS [9]. The third document provides for a certification, but this is only voluntary.

## 5. Cyber-Attacks Applicable to Care Robots

**Figure 2:**
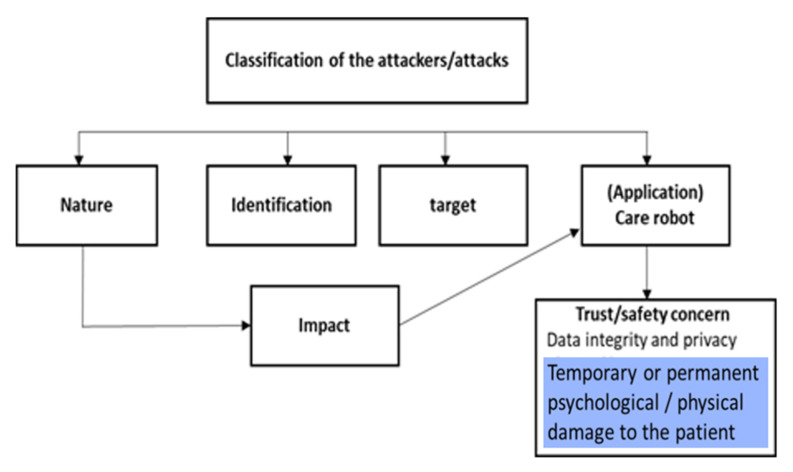
Model of robot-related threats, causes, and consequences.

CS for CRs must consider a broader spectrum of problems than other critical MDs, where, nevertheless, CS is more consolidated, such as the pacemakers [57,58,59] and the artificial pancreas [60,61,62]. CRs can generate, for example a psychological harm (Figure 1). This is also a consequence of issues dealt in par. 3 [30,31]. Much of the experience in robotics [16,17,18,19,20] on physiological harms/damages can be exported to CRs. Indeed, in [21] a process of unification has been carried out, which has general validity. Figure 2 summarizes the different robot-related threats, their causes, and their consequences in the case of the CRs. With reference to the figure, the nature of the attack is: internal vs external, coordinated vs random, detected/undetected, corrected/uncorrected. The identification is: data confidentiality and privacy, message authentication, device/user authentication, system integrity, data availability, system availability. The target is: the application layer, the hardware layer, the firmware layer. The impact can be low, moderate, high. The trust and safety concerns (according to the model in paragraph. 2) are data integrity and privacy, physical harm, physical damage, psychological harm.

The Attacks can be arranged into three categories [21]: ATTACKs on the hardware, ATTACKs on firmware, ATTACKs on the communication. In the following, we summarize these categories in brief.

### 5.1. Attacks on the Hardware

These ATTACKs [21] vary from hardware Trojans up to phishing [63]. They allow the aggressor to create passages to gain unauthorized access up a full control [21,64]. In some cases, they can even have a full access to the hardware. We can also find the implementation ATTACKs or fault ATTACKs [64]. These are very dangerous and can cause to sensitive data damage or system corruption.

### 5.2. Attacks on the Firmware

**Figure 3:**
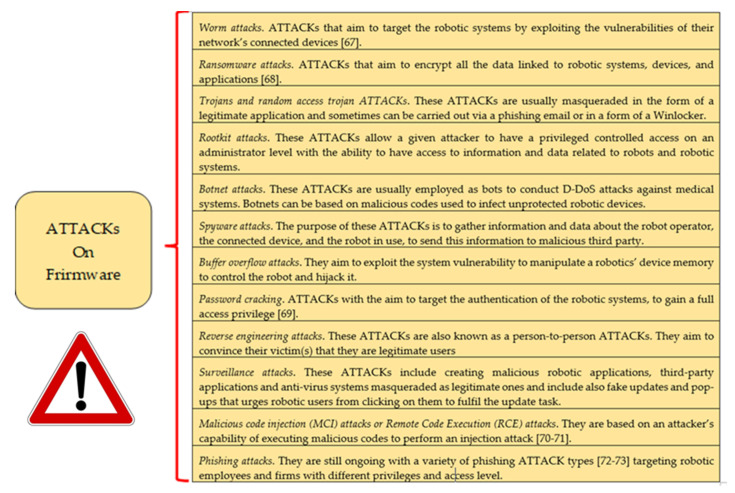
Examples of ATTACKs on the firmware.

According to [21,65,66], as the OS upgrading/maintenance is mainly performed using the internet, the OS is exposed to DoS and D-DoS ATTACKs, along with the indiscriminate programme execution, and root-kit ATTACKs. Furthermore, the Applications in the CRs, are vulnerable to application ATTACKs. These ATTACKs comprehend malware, worms, viruses, software Trojans ATTACKs, buffer overflow, and malicious code injection ATTACKs [67]. Figure 3 reports examples of these ATTACKs [21,67,68,69,70,71,72,73]:

### 5.3. Attacks on Communications

**Figure 4:**
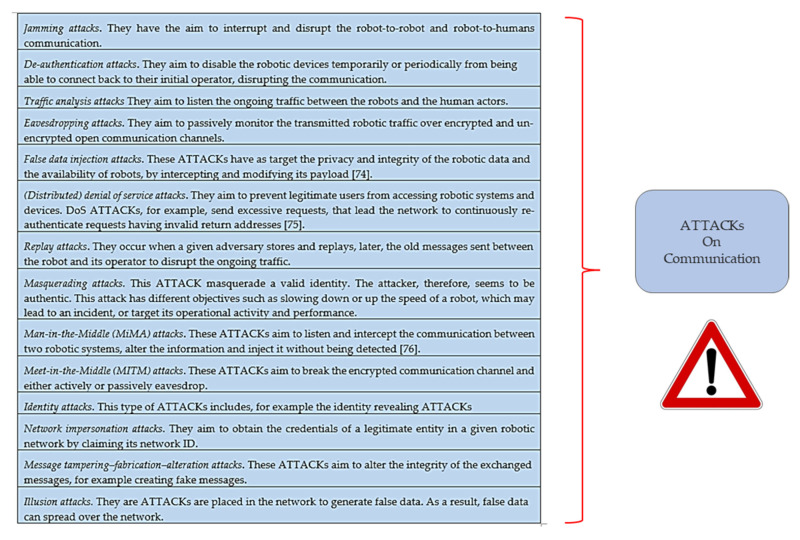
Examples of ATTACKs on the communication.

Robotic communications are also exposed to different ATTACKs [21,74,75,76,77] that can affect different levels of security at different levels of communication (Figure 4):

## 6. Conclusions

### 6.1. Highlights

CRs [7] represent an opportunity for the health domain. The use of these robots has important implications. They can be used in surgery [7], in important and delicate clinical interventions both in presence and in tele-surgery. They can be used on frail patients, in rehabilitation processes [8]. They can be used in psychological and cognitive rehabilitation processes, as in the case of SRs, in children, elderly, and other subjects with disabilities [3,4]. Therefore, they have important physical and psychological implications during their use [9]. Furthermore, these devices, during their use, encounter important demographic-and-clinical data and other reserved information; all data that must be protected, in accordance with current regulations [1,2]. CS has consequently become a crucial issue. It concerns all the actors involved (from the design process to its use; from the manufacturer up to the patient and the caregiver). The study investigated the collocation of CRs in the context of CS studies in the health domain, also in comparison to other sectors. Problems and peculiarities were faced, investigating in different scientific database. They ranged from ethics and safety up to legislation and regulation issues.

The highlights of the study are as follows:

- A simple search on the Pubmed database, the most important database of the health domain, shows 12.785 results on the CS [12]. Among these, an important group [13] is dedicated to robotics. However, many studies on robotics linked to CS can be traced with the other keys safety and risk [14,15].
- CRs have peculiarities that make them unique. However, regarding some issues, the experience of robotics used in the IPCS robotics can be partly taken into consideration [16,17,18,19,20].
- CRs are complex mechatronic tools, but also HCI and devices integrated to eHealth [27,28,51]. Scientific support come also from both initiatives of scientific societies, operating in these sectors [27] and proper approaches on the insiders [28].
- Ethics has an important role and a peculiarity on CRs, such as on the SRs [29]. An in-depth analysis of the ethical issues in this discipline has identified two macro-sectors [30,31]. The first macro-sector is the ethics in a responsible research and innovation [30]. The second macro-sector is the ethics problem encountered while building moral CRs [31]. A strong connection emerges between ethical issues and CS from the examination of the two macro-sectors (also correlated). There is a strong need to rethink a CS connected to ethics issues.
- The models between the Cyber ATTACKs/ Software default/AI deficits and the physical/ psychological impact, have been identified [9]. They also embed the problems identified in the previous point [30,31]. These models show a wider range of CS problems than other consolidated MDs [57,58,59,60,61,62].
- Cyber ATTACKs applicable on the CRs, and the related impact, have been identified and categorized into three groups [21] concerning hardware [63,64], firmware [65,66,67,68,69,70,71,72,73], and communication [74,75,76,77].
- Targeted surveys with interviews and questionnaires regarding the CS behaviours of insiders with CRs will have to be conducted, as already been carried out, for example, in the health domain generally [28]. This will be useful for building medical knowledge.
- There are shadows in EU MD regulations [53]. First, it focuses a lot on manufacturers and little on recipients/ users. Second, [9] the compliance with CS requirements is challenging, in part due to the potential overlap of different certification schemes with varying geographical or product scope and evolution of external to the MDR regulations. Third, the intended use and certification, often, do not seem aligned.
- There are limits in the application of specific CS certifications. They are voluntary, as in the case of the Cybersecurity ACT [56].
- The CRs would need an ad hoc regulatory framework, in consideration of the peculiarities.

### 6.2. Reflections

We believe that, in the light of what is covered in our study, it is important to plan an acculturalization process on CS, with specific reference to CRs. This process must concern all the involved actors, from the builders up to the users, and the caregivers. It must be conducted in the different environments (e.g., home and the hospital). Training in this area must become an important issue. In addition, agreement initiatives (e.g., guidelines, consensus conferences, and technology assessment initiative [78,79,80,81,82,83,84]) considering CS could be welcome. Stakeholders will have to take actions in this area, through consensus initiatives (for example, considering the CS in consensus conferences), specific monitoring initiatives (for example through targeted surveys), and specific interventions on the training.

## Figures and Tables

**Table 1:** Classification of the rehabilitation robot according to the applications.

Application	Description
Upper limb rehabilitation	Allowing rehabilitation of the upper limb using exoskeletons or end-effector system
Lower limb rehabilitation	Allowing rehabilitation of the lower limb using exoskeletons or end-effector system
Stability	Allowing the stability training and recovery using exoskeletons or end-effector system

## Data Availability

Not applicable.
